# Deciphering the Association among Pathogenicity, Production and Polymorphisms of Capsule/Melanin in Clinical Isolates of *Cryptococcus neoformans* var. *grubii* VNI

**DOI:** 10.3390/jof8030245

**Published:** 2022-02-28

**Authors:** Nórida Vélez, Nelson Vega-Vela, Marina Muñoz, Paola Gómez, Patricia Escandón, Juan David Ramírez, Oscar Zaragoza, Lucía Monteoliva Diaz, Claudia-Marcela Parra-Giraldo

**Affiliations:** 1 Unidad de Proteómica y Micosis Humanas, Grupo de Enfermedades Infecciosas, Departamento de Microbiología, Facultad de Ciencias, Pontificia Universidad Javeriana, Bogotá 110231, Colombia; velez.norida@javeriana.edu.co (N.V.); vega.nelson@javeriana.edu.co (N.V.-V.); biopagoru@gmail.com (P.G.); 2 Centro de Investigaciones en Microbiología y Biotecnología-UR (CIMBIUR), Facultad de Ciencias Naturales, Universidad del Rosario, Bogotá 111221, Colombia; claudia.munoz@urosario.edu.co (M.M.); juand.ramirez@urosario.edu.co (J.D.R.); 3 Grupo de Microbiología, Instituto Nacional de Salud, Bogotá 111321, Colombia; pescandon@ins.gov.co; 4 Molecular Microbiology Laboratory, Department of Pathology, Molecular and Cell-Based Medicine, Icahn School of Medicine at Mount Sinai, New York, NY 10029, USA; 5 Mycology Reference Laboratory National Centre for Microbiology, Instituto de Salud Carlos III, 28222 Madrid, Spain; ozaragoza@isciii.es; 6 Departamento de Microbiología y Parasitología, Facultad de Farmacia, Universidad Complutense de Madrid, 28040 Madrid, Spain; luciamon@ucm.es

**Keywords:** *Cryptococcus neoformans*, MLST, polymorphism, capsule, melanin

## Abstract

Background: *Cryptococcus neoformans* is an opportunistic fungal pathogen that can cause meningitis in immunocompromised individuals. The objective of this work was to study the relationship between the phenotypes and genotypes of isolates of clinical origin from different cities in Colombia. Methods: Genome classification of 29 clinical isolates of *C. neoformans* var. *grubii* was performed using multilocus sequence typing (MLST), and genomic sequencing was used to genotype protein-coding genes. Pathogenicity was assessed in a larval model, and melanin production and capsule size were evaluated in vitro and in vivo. Results: Eleven MLST sequence types (STs) were found, the most frequent being ST69 (*n* = 9), ST2, ST93, and ST377 (each with *n* = 4). In the 29 isolates, different levels of pigmentation, capsule size and pathogenicity were observed. Isolates classified as highly pathogenic showed a tendency to exhibit larger increases in capsule size. In the analysis of polymorphisms, 48 non-synonymous variants located in the predicted functional domains of 39 genes were found to be associated with capsule size change, melanin, or pathogenicity. Conclusions: No clear patterns were found in the analysis of the phenotype and genotype of *Cryptococcus*. However, the data suggest that the increase in capsule size is a key variable for the differentiation of pathogenic isolates, regardless of the method used for its induction.

## 1. Introduction

Cryptococcosis is an opportunistic fungal infection that affects humans and animals, and it is believed that the infection occurs via the inhalation of fungal propagules from an environmental source [1]. This mycosis is potentially fatal in immunocompromised individuals, and compromises the respiratory system and the central nervous system. This infection is caused by a complex of *Cryptococcus neoformans* and *Cryptococcus gattii* species [2]. Although in 2015, Teun Boekhout et al. proposed the classification of *C. neoformans* and *C. gattii* into seven species, in the present investigation, we will describe the complex of the two species as described earlier by Kwon-Chung [3,4,5].

By 2014, the annual incidence of cryptococcosis worldwide was 223,100 cases [6]. In South America, Colombia is the country with the second highest number of reports of cryptococcosis [7]. In the period of 1997–2016, the annual incidence was 1.1 cases per 1000 people living with HIV/AIDS, and 0.23 cases per 100,000 people in the general population. The species with the highest frequency isolated in Colombia is *C. neoformans*, accounting for 96.3% of isolates, and the most prevalent molecular type is VNI, at 96.1% [8]. This molecular type is also the most frequently isolated worldwide in clinical and environmental samples [7], followed, to a lesser extent, by VNII [9], VNIII, and VNIV. These last two patterns are frequently reported in Europe [10].

*C. neoformans* var. *grubii*, H99 corresponds to the VNI genotype and to multilocus sequence typing (MLST) sequence type (ST) two, and its genome sequence is available [11,12,13]. Inter- and even intra-laboratory variability of this strain have been reported, revealing microevolutionary processes that impact both the genotype and the pathogenic performance of this strain [14]. Similar variability has been observed in clinical isolates [15]. Therefore, it is of interest to study the variability among clinical isolates to understand both the genetic and the environmental factors that govern the pathogenic capacity of this fungus, including molecular patterns that may allow its performance to be predicted.

The pathogenic capacity of the fungus results from the expression of virulence factors that contribute, in a multifactorial way, to the ability of the yeast to establish a niche in the susceptible host. This includes both escaping the immune system and establishing itself as a parasite. In this context, the main virulence factors are the production of an inducible capsule that can be adapted to various situations [16], as well as the modulatable production of melanin [17].

The capsule is anchored to the cell wall. Among its essential functions are those inhibiting the outcomes of oxidative stress in the phagosome and allowing the fungus to establish itself as a facultative intracellular pathogen, playing a key role in escaping phagocytosis [18]. The capsule varies in size during infection among strains and individual cells, depending on the environmental conditions in which the fungus is found. It has been shown that an increase in capsule size can be induced by high CO_2_ levels, low iron levels, basic pH values, and the presence of mammalian serum [19]. Melanin inactivates intracellular death mechanisms, protects the fungus against oxidants generated by host cells, and prevents the production of tumor necrosis factor (TNF) by macrophages, which is necessary to generate a protective cellular immune response [20,21,22]. Furthermore, melanin has been reported to protect *Cryptococcus* against the action of antifungals, such as amphotericin B and azoles [20,21].

Research on *C. neoformans* as an organism of clinical importance has addressed its molecular and genetic epidemiology, as well as identifying genes associated with the main virulence factors (capsular polysaccharides and melanin). In epidemiological studies, various molecular approaches have been used, including PCR fingerprinting, restriction-fragment-length polymorphisms (RFLPs), amplified-fragment-length polymorphisms (AFLPs), and MLST. The latter also allows traceability at a global level through sequencing and assignment of STs on the basis of seven genes (*CAP59*, *LAC1*, *GPD1*, *SOD1*, *PBL1*, *URA5*, and *IGS1*) [23].

Great advances have been made in the genome sequencing of *C. neoformans*, and annotated genome assemblies of reference strains of serotype A (H99/125.31), serotype D (JEC20/JEC21) and serotype B (WM2276/E566) are available. The *C. neoformans* genome is approximately 20 megabases in length, is organized into 14 chromosomes, has abundant introns, and contains approximately 6500 genes [11,12,13,14]. 

Genome analysis has allowed the identification of genes involved in capsule biosynthesis, such as the *CAP* gene family (*CAP10*, *CAP59*, *CAP60* and *CAP64*) [24,25]. Similarly, both experimental and in silico approaches have allowed the identification of genes with functions related to the growth of the capsule or to routes and transduction signals that modulate its synthesis [18,19]. There are also external factors that implicate capsule biosynthesis, such as iron levels in the environment through *CIR1*, the response to different pH levels through Rim101 and the stress response involving the Hog1 and Pka1/cAMP pathways [26].

Likewise, genes that are activated to produce melanin in the presence of exogenous diphenolic compounds, such as l-3,4-dihydroxyphenylalanine (l-DOPA) have been identified. *C. neoformans* produces eumelanin through laccases (Lac1 and Lac2), and the regulation and production of this pigment involve the activation of cAMP and Hog pathways; the four transcription factors Bzp4, Usv101, Hob1 and Mbs1; and the central kinases Gsk3 and Kic1. Laccases are loaded into secretory vesicles and deposited as particles within the cell wall using chitin as an anchor molecule, or are secreted extracellularly [27,28,29,30,31].

In view of this scenario, the present study focused on *C. neoformans* var. *grubii* isolates of clinical origin from different cities in Colombia. The STs were determined by MLST, the pathogenic capacity and main virulence factors were characterized, and the variation in these phenotypes among isolates and STs was then compared to the variation in the corresponding genes using a whole-genome sequencing approach. In this analysis, polymorphisms of genes associated with biosynthesis and/or the regulation of capsule or melanin were determined. We reasoned that these approaches would allow us to identify patterns that could lead to an improved understanding of variability in the pathogenic performance of this fungus.

## 2. Materials and Methods

### 2.1. Biological Material and Demographic and Clinical Data

A total of 29 clinical isolates of *C. neoformans* var. *grubii* were selected from the collections of the Microbiology Group of the National Institute of Health, Bogotá, Colombia. Each isolate was typed and confirmed by PCR fingerprinting and restriction-fragment-length polymorphism (RFLP) of the *URA5* gene.

The clinical isolates were from six departments in Colombia (Atlántico, Antioquia, Cauca, Meta, Norte de Santander, and Valle), 73.9% of which were isolated from patients of the male sex, with an average age of 41 years (min.: 21; max.: 72). The most common symptom was headache (87%), followed by vomiting (65.2%), fever (60.9%), and to a lesser extent, confusion, visual disturbance, cough, and weight loss. A total of 86.9% of the cases presented some risk factor, of which the most common was HIV/AIDS (82.6%); 21.7% had AIDS when cryptococcosis was diagnosed, and 21.7% of the patients died (Tables S1 and S2).

### 2.2. Assessment of Pathogenicity of Isolates in Galleria mellonella

*Galleria mellonella* larvae were obtained from a Scientia breeding facility (Cali, Colombia). Late-stage larvae (fifth and sixth) with weights between 250 and 330 mg and a length of approximately 2 cm were chosen. Procedures were carried out as described by Velez et al., 2018 [32]. The isolates were cultured in 2% Sabouraud dextrose broth (SDB) at 35 °C and 110 rpm for 15 h, and the inoculum was adjusted to 1.5 × 10^8^ cells/mL using a Neubauer chamber. Inoculation of the larvae was performed with 10 µL of yeast suspension by injection into the last left proleg of larvae using a 0.5 mL BD syringe. After puncture, the larvae were incubated in Petri dishes at 37 °C, and the number of dead larvae was recorded daily for 15 days.

A group of 50 larvae was implemented as an absolute control (uncleaned, uninoculated), followed by disinfection (cleaned with ethanol), inoculation (received 10 μL sterile phosphate-buffered saline buffer (PBS)), control strains of high (H99: *C. neoformans* var. *grubii*) and low (JEC21: *C. neoformans* var. *neoformans*) pathogenicity groups. To compare mortality in the invertebrate model, 10 specimens were used for each control and for each isolate. Three biological replicates were made, for a total of 30 larvae for each isolate.

### 2.3. Capsule Size Determination

#### 2.3.1. Effect of the Change in Capsule Size Pre- and Post-inoculation in *G. mellonella*

Isolates were cultured in 2% SDB for 15 h at 110 rpm at 37 °C, and inoculum was adjusted to 1 × 10^5^ cells/mL using a Neubauer chamber; next, a microscopic slide containing one drop of India ink and one drop of each strain was prepared and visualized on a Zeiss Axiophot Microscope (Carl Zeiss B.V., Breda, The Netherlands) at 80×. Capsule size was defined as the difference between the size of the whole cell (yeast cell + capsule) and the size of the cell body (as limited by the cell wall), and 50 cells were measured for each isolate. To perform these measurements after inoculation in the larvae, strains were recovered from *G. mellonella* after the survival assay. Each dead larva was macerated and homogenized in 1 mL of PBS, and the procedure previously described was used to measure capsular size [33].

#### 2.3.2. Capsule Induction

Capsule induction was performed following the protocol described by Zaragoza et al. using capsule-inducing medium (10% SDB in 50 mM MOPS, pH 7.3) [34]. An inoculum was adjusted to 1 × 10^8^ cells/mL prior to plating. The cultures were incubated overnight at 37 °C with shaking (110 rpm). Following microscopy observation, a microscope slide containing one drop of India ink and one drop of each isolate was prepared and observed using a Leica DMI 3000B microscope (Leica Microsystems, Wetzlar, Germany). Images were acquired in bright field with 40× or 63× objectives, and capsule size and cell body (delimited by cell wall) were measured in 50 cells using ImageJ Software (Fiji) V2.1 [35]. The capsule area of 50 cells was measured for each isolate, and the procedure previously described was used to measure capsule size.

### 2.4. Melanin Production

The isolates were seeded in 2% SDB at 37 °C for 24 h with shaking at 110 rpm; subsequently, the inoculum was adjusted to 2 × 10^7^ cells/mL in PBS. Serial 1:10 dilutions were performed, and 5 μL from each dilution were spotted in chemically defined minimal medium (15 mM dextrose, 10 mM MgSO_4_, 29.4 mM KHPO_4_, 13 mM glycine, 3 μM thiamine, pH 5.5) supplemented with 1 mM l-DOPA (Sigma-Aldrich, St. Louis, MO, USA), and with 1.5% agar powder. The plates were incubated at 37 °C and protected from light. Pictures were taken daily for 5 days.

### 2.5. Genome Sequencing and Data Preprocessing

We sequenced the genomes of 29 clinical isolates to determine similarities and/or dissimilarities at the genomic level, and to investigate whether there is an association between virulence factors, particularly capsule and melanin production, and polymorphisms in genes known to be involved in their production. To this end, we cultured each clinical isolate, which had been stored at −80 °C since collection, in 2% SDA at 37 °C for 48 h. Then, one colony was inoculated on yeast-extract–peptone–dextrose agar (YPD) at 37 °C for 24 h. One hundred microliters of cultured yeast cells were used for DNA extraction using the UltraClean^®^ Tissue & Cells DNA Isolation Kit (MoBio Laboratories Inc., Carlsbad, CA, USA) with the standard protocol recommended by the manufacturer. DNA integrity was checked on a 1% agarose gel and visualized with SYBR Safe DNA stain (Invitrogen, Waltham, MA, USA).

Neither DNA degradation nor RNA contamination was detected. The quality and quantity of isolated DNA was examined and measured using a NanoDrop2000 spectrophotometer (Thermo Scientific, Waltham, MA, USA) [36]. Then, 2 × 150 bp paired-end sequencing was performed using the Illumina HiSeq platform at Novogen Bioinformatics Technology Co., Ltd. (Beijing, China). Raw sequencing data were assessed with FastQC v0.11.9 [37], and potential contaminants were identified and discarded. The raw sequence reads were mapped against the H99 reference genome of *C. neoformans* var. *grubii* (RefSeq assembly accession: GCF_000149245.1) with Bowtie2 version 2.3.5.1 [38], and a BLASTn search [39] was performed against the complete bacterial and fungal RefSeq database (downloaded 20 April 2020) with a minimum e-value of 10e-^12^ using BLAST+ 2.10.0+ [40]. Finally, we cleaned the filtered sequences, trimming Illumina adapters and low-quality bases (with a minimum Phred score threshold of 20), and removing short read sequences (<50 bp) using fastp ver. 0.20.0 [41] The raw sequence data were deposited in the NCBI Sequence Read Archive (https://www.ncbi.nlm.nih.gov/bioproject/?term=PRJNA669191, accessed on 11 September 2021) under BioProject ID number PRJNA669191.

### 2.6. Multilocus Sequence Typing

We typed 29 clinical isolates by MLST to establish their molecular type (MT) and ST in accordance with the International Society for Human and Animal Mycology (ISHAM) consensus MLST scheme for the *C. neoformans*/*C. gattii* species complex [23]. The allele sequences of all MLST loci were retrieved from the set of predicted protein-coding genes or contigs.fasta files for each clinical isolate, which included the following genetic loci: capsule polysaccharide (*CAP59*), glycerol 3-phosphate dehydrogenase (*GPD1*), laccase (*LAC1*), phospholipase B1 (*PLB1*), superoxide dismutase (*SOD1*), orotidine monophosphate pyrophosphorylase (*URA5*) genes, and the ribosomal intergenic spacer (*IGS1*) region. Allele type (AT) and ST identification were performed by pairwise alignment using the pairwise ID algorithm and the polyphasic ID algorithm of BioloMICS, respectively, implemented in the ISHAM *C. neoformans* MLST database server (http://mlst.mycologylab.org, accessed on 11 September 2021). The allelic profiles retrieved determined the corresponding sequence types.

The genetic diversity of the isolates was determined using DnaSP v5 software from concatenated sequences of the isolates to detect levels of genetic polymorphism. The distribution was determined by calculating the diversity of haplotypes (genes), the diversity of nucleotides (π) (the average number of differences of nucleotides per site between two sequences), and the indices θ (per site, as an indicator of the rate of mutation by nucleotide site per generation), and calculated from Eta (h) (the total number of mutations, and “S”, the number of polymorphic segregating sites) [42].

### 2.7. Identification and Analysis of Genetic Variants

To study genetic variability among clinical isolates of *C. neoformans* var. *grubii*, genetic variants were called and analyzed with the next-generation sequencing expertise platform—NGSEP ver. 4.0.1 [43]. NGSEP performs accurate detection and genotyping of single nucleotide variants (SNPs), small and large insertions/deletions (indels), short tandem repeats (STRs), inversions, and copy number variants (CNVs). Only genetic variants that mapped to genes related to capsule and melanin were considered for this SNP study.

Initially, the processed sequence reads for each clinical isolate were mapped to the H99 reference genome using the ReadsAligner NGSEP module, then mapped according to their location in the reference genome with Picard Tools [44]. The NGSEP MultisampleVariantsDetector Module was then used to detect genetic variants for multiple samples. This module was run with the arguments -psp, -embeddedSNVs, -ploidy 1, maxAlnsPerStartPos 30, -maxBaseQS 30, and -minQuality 20.

The VCFAnnotate NGSEP module was used for variant annotation applying the H99 reference genome annotation and the VCFFilter NGSEP module to filter genetic variants with the arguments -fi, -fir, -m 1, -q 30, and -minRD 3. Genetic variants corresponding to multiallelic states and/or heterozygous sites were filtered.

Genetic distance matrices were calculated with the VCFDistanceMatrixCalculator NGSEP module using the basic IBS (identity by state) algorithm. Genomic differences among the clinical isolates were analyzed by constructing a dendrogram with the NeighborJoining NGSEP module. The trees were visualized and edited with Dendroscope ver. 3.7.2 [45].

Were sought to look for associations between the main virulence factors, associated genes and pathogenicity obtained from the *G. mellonella* model. To achieve this, we initially performed a principal component analysis of the main virulence factors and pathogenicity. Finally, the genetic relationships between the clinical isolates were also examined via PCA2 using the R package SNPRelate version 1.12.2 [46]. The ld.threshold argument was set to 0.99 of the snpgdsLDpruning function, reducing the number of variants by 40% on average. Whether the proportions of a nominal genotypic variable (e.g., biallelic states) were different between values of a phenotypic variable (e.g., capsule size groups) was determined using Fisher’s exact test of independence or post hoc pairwise Fisher’s exact test (Figure S1).

Subsequently, genes of *C. neoformans* that are known or suspected to be associated with, or related to, its virulence factors were retrieved in PHI-base, the virulence factor database in Fungal Pathogens -DFVF, the Victors database, and previously published studies. Finally, the genetic variants detected in the genomic analysis, associated with the production of virulence factors, and present in the virulence factor dataset were analyzed and contextualized using FungiDB, virulence factor databases and other bioinformatics resources for functional annotation.

### 2.8. Statistical Analysis

For numerical variables, measures of central tendency were applied, and for categorical variables, the Chi-square test or Fisher’s exact test was used; the results were considered statistically significant with *p*-values of less than 0.05. Stata software Version 11.1 was used. The capsule results were compared using one-way analysis of variance (ANOVA), applying Levene’s test of homogeneity of variance and Bonferroni’s test of multiple comparisons. Survival curves were constructed using the Kaplan–Meier method, then the curves were compared using the log-rank (Mantel–Cox) test. Statistical analysis was performed using GraphPad Prism 5 software. Images obtained by microscopy were analyzed in the Fiji ImageJ program [35].

## 3. Results

### 3.1. Identification of the Sequence Types of the Clinical Isolates of C. neoformans var. grubii

A total of 29 clinical isolates of *C. neoformans* var. *grubii* obtained from seven cities were chosen from the strain collection of the National Institute of Health in Colombia. Using MLST, the STs of the isolates were identified using the consensus of the ISHAM for the species complex *C. neoformans*/*C. gattii* [23].

**Figure 1:**
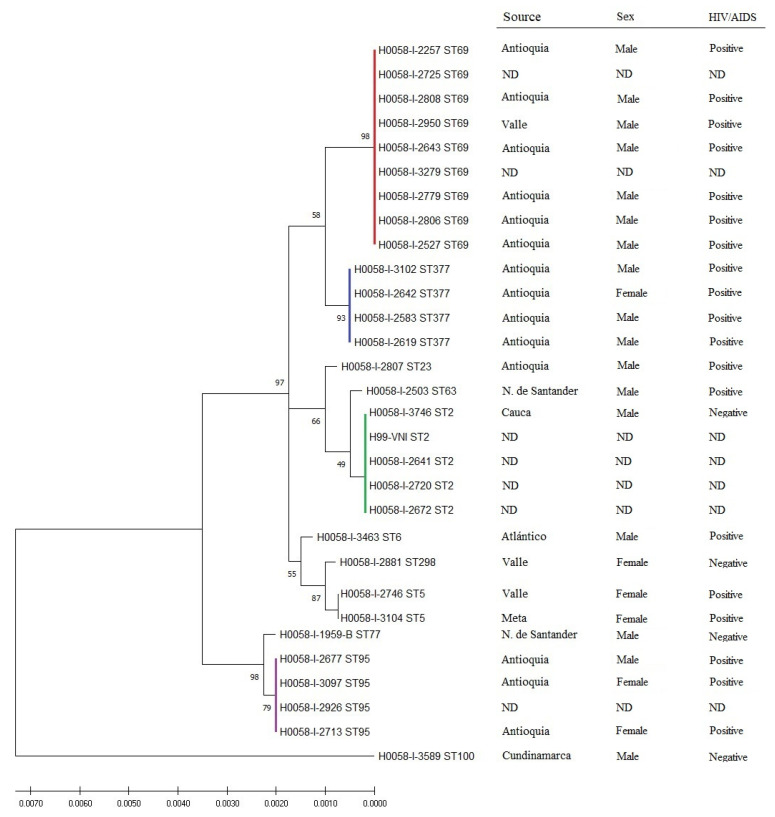
Hierarchical grouping of the 29 clinical isolates of *C. neoformans* var. *grubii* based on the MLST allelic profile. The figure shows the relationship among the STs of the clinical isolates. The evolutionary history, as inferred from the MLST data, was reconstructed via the maximum likelihood method and the Jukes–Cantor model using concatenated nucleotide sequences of 7 loci. Bootstrap values are displayed for each branch (1000 repeats). Representative clinical variables are listed on the right of the image. The most frequent sequence types are highlighted in colors. ND: no data.

In the 29 isolates, 2 molecular types and 11 sequencing types were identified, namely, VNI (28 isolates) and VNII (1 isolate; Table S2). Regarding the latter, isolate 3589—characterized as ST100 and MT VNII—had been previously classified by fingerprint PCR as VNI. The distribution of the represented and allelic profiles was dominated by ST69, which accounted for nine of the twenty-nine isolates (frequency 0.3103), followed by ST2, ST95 and ST377, each of which contributed four of twenty-nine isolates (each with frequency 0.1379). The least frequent STs were ST5 with two isolates (frequency 0.069) and ST6, ST23, ST63, ST77, ST100 and ST298, each with one isolate (frequency 0.0345). The basic genetic classification and relationship among the *C. neoformans* isolates, as deduced solely from MLST, are shown in Figure 1, Tables S2 and S3. The number of MLST haplotypes was 10, the nucleotide diversity index was 0.860, the number of polymorphic sites was 70, the diversity of nucleotides was 0.00311, and the θ index was 0.00443.

### 3.2. Assessment of Pathogenicity in G. mellonella

To determine the pathogenicity profile of clinical isolates of *C. neoformans* in *G. mellonella*, two controls (reference strains) with high (H99) and low (JEC21) pathogenicity were included [47]. High or low pathogenicity was assessed via the speed at which the isolate led to the death of the larvae after infection (days post-infection). In the highly pathogenic reference strain H99, death occurred approximately 3 days after infection, and in the weakly pathogenic reference strain JEC21, death occurred at approximately 7 days.

**Figure 2:**
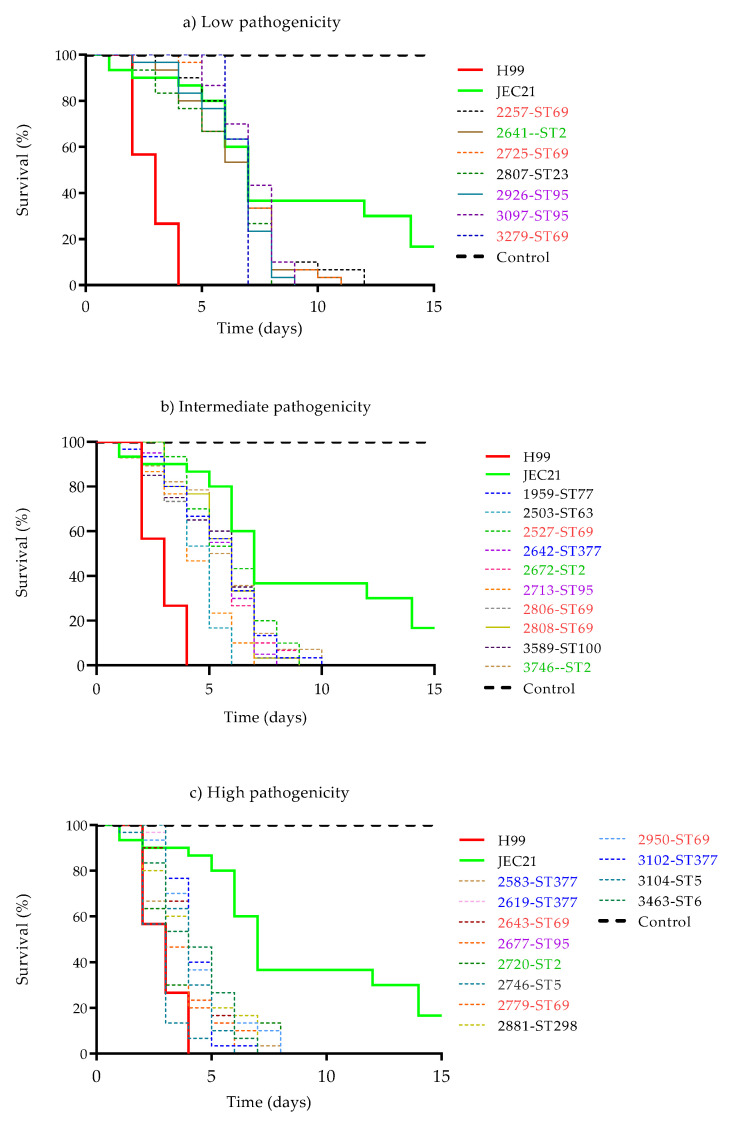
Survival curves of the 29 clinical isolates of *C. neoformans* with (**a**) low, (**b**) intermediate, and (**c**) high pathogenicity in *G. mellonella.* The most frequent STs are shown in green (ST2), red (ST69), purple (ST95) and blue (ST377). After injection with 1.5 × 10^8^ cells/mL, Curves were constructed using the Kaplan–Meier method, and then the curves were compared using the log-rank (Mantel–Cox) test. The data are expressed as survival percentages. No larval death was observed in control larvae injected with an equivalent volume of PBS.

The 29 Colombian isolates spanned the range between these two extremes. A comparison of survival curves and average survival times obtained in three biological replicates showed that seven of the twenty-nine isolates presented low pathogenicity with a median survival of 6 to 7 days (*p* < 0.0001), ten of twenty-nine presented intermediate pathogenicity with a median survival of 4 to 5 days, and twelve of twenty-nine presented high pathogenicity with a median survival of 2 to 3 days (*p* < 0.0001) (Figure 2 and Table S4). The relationship of pathogenicity in *G. mellonella* to the MLST results and demographic variables was then analyzed.

*Pathogenicity—MLST*. The seven isolates with low pathogenicity belonged to four of the 11 STs, the ten isolates with intermediate pathogenicity belonged to seven STs, and the twelve isolates with high pathogenicity belonged to seven STs (Figure 2). Mortality in larvae caused by infection by ST69 isolates varied from 3 to 7 days post-infection, and mortalities for ST2 isolates varied from 2 to 6 days, for ST95 from 3 to 6 days and for ST377 from 3 to 5 days.

*Pathogenicity—demographic data*. Of the four out of the seven isolates categorized with low pathogenicity for which provenance was known, they all came from Antioquia and from HIV/AIDS-positive patients. The isolates categorized with intermediate pathogenicity came from four departments (Antioquia, Cundinamarca, Cauca, and Norte de Santander), of which 40% were from HIV/AIDS-positive patients. Finally, the isolates categorized as having high pathogenicity also originated in four departments (Antioquia, Atlántico, Meta, and Valle), and 91.6% were obtained from HIV/AIDS-positive patients (Table S2).

### 3.3. Determination of Capsule Size Change In Vivo Using the G. mellonella Invertebrate Model

We recorded the morphological change of the capsule during infection of each of the 29 Colombian isolates in *G. mellonella*. To determine the post-inoculation capsule size, yeast was recovered on the day that 50% of the population was alive, which depended on the isolate.

For the 29 isolates evaluated, the average of the capsule in the yeasts before inoculation was 0.93 µm (σ = 0.365), and post-inoculation, it was 2.21 µm (σ = 0.954). In 25 of 29 isolates, statistically significant differences in capsular size (*p* < 0.0001) were observed when all three biological replicates were included. Next, we analyzed the relationships of the change in capsular size after inoculation in *G. mellonella* with the previous results of pathogenicity and with the MLST classification (Table S2).

*Capsule size—pathogenicity*. When comparing the isolates categorized as having low and high pathogenicity according to the results obtained in *G. mellonella*, we observed that capsule size increased substantially in 27/29 isolates after the yeast passed through the larvae. In the isolates classified as having low pathogenicity, we observed capsules with an average diameter of 1.49 µm (σ = 0.423) and, in the group with high pathogenicity, 1.42 µm (σ = 1.272) (Figure S2).

**Figure 3:**
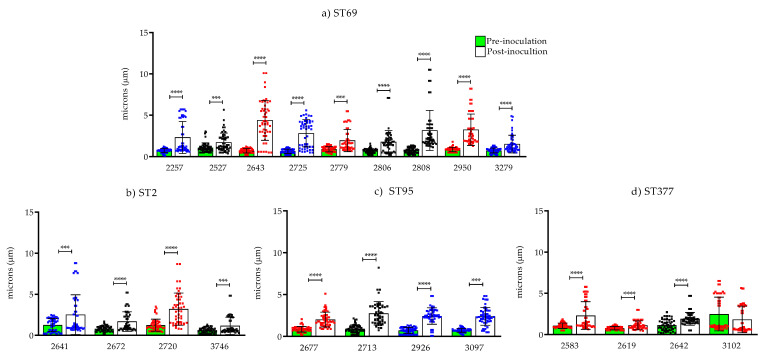
Capsule size pre- and post-inoculation in *G. mellonella* of the 21 clinical isolates of *C. neoformans* var. *grubii* belonging to the most frequent sequence types: (**a**) ST69, (**b**) ST2, (**c**) ST95, and (**d**) ST377. Boxes are colored according to isolates’ pathogenicity: blue (low), black (intermediate) and red (high). The error bars represent the standard deviation. *p* < 0.001 (***); *p* > 0.0001 (****).

*Capsule size—MLST*. When analyzing the relationship of capsular sizes with the most frequent STs (*n* = 21 isolates), we observed that ST2, ST69 and ST95 presented a greater increase in capsular size (1.158, 1.755 and 1.601 µm, respectively) than the isolates from ST377, which presented less change (0.425 µm) (Figure 3). There was no statistically significant association between pre- and post-induction capsular size in *G. mellonella* and the results obtained in the pathogenicity assessment (*p* = 0.965) or ST (*p* = 0.216).

### 3.4. Capsule Induction

Capsule growth was also induced in vitro by capsule induction medium in the 29 isolates (see Section 2.3.2). In 22 isolates, the capsules increased significantly with respect to the basal conditions (*p* < 0.0001)—the mean of the capsule of the 29 isolates being 1.03 µm in basal conditions (σ = 0.240) and 1.91 µm (σ = 1.681) after induction by capsule induction medium—where these results summarize the three biological replicates.

We found generally good agreement (*R* = 0.83) between isolates’ capsule size changes seen in vitro (from basal to induction capsule size) and the same isolates’ capsule size changes we observed in vivo (from pre- to post-inoculation) in *G. mellonella*. For the other phenotypic characteristics, we analyzed the relationship of capsular growth induction with pathogenicity and MLST profiles (Table S2).

**Figure 4:**
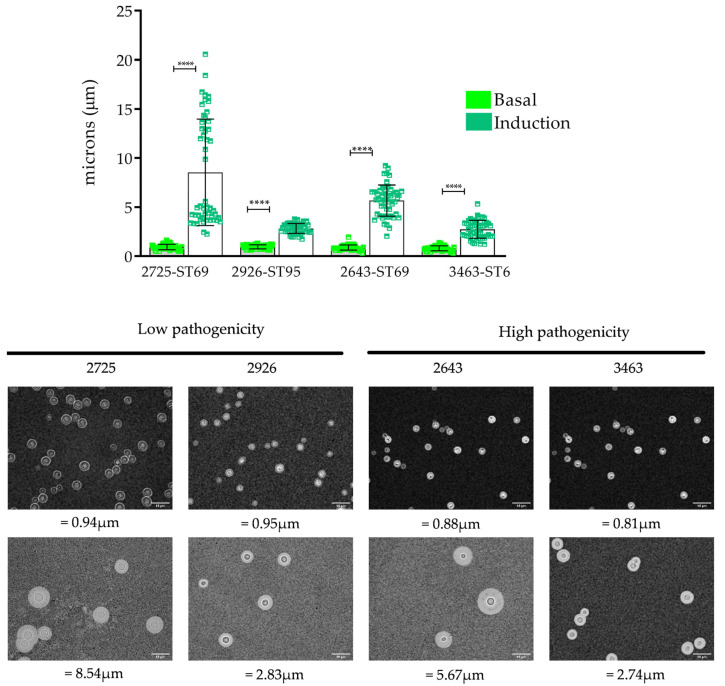
Capsule induction in vitro; figure constructed using the isolates with the greatest changes in capsule size. Bright green represents the basal capsule size, and dark green represents the size after capsule induction. The bottom figure shows the photographic record of the isolates. *p* < 0.0001 (****). The scale at the bottom right of the images represents 10 µm.

*Capsular induction—pathogenicity*. In the seven isolates we classified as having low pathogenicity, larger capsules were observed after induction (1.730 µm on average) compared to the isolates with high (0.630 µm) and intermediate (0.498 µm) pathogenicity (Figure S3). The largest capsule sizes were observed in four isolates, with two and two isolates showing high and low pathogenicity, respectively (Figure 4). The isolate that presented the highest capsule growth was isolate 2725, categorized as having low pathogenicity. On average, the capsule was 0.94 µm under basal conditions and 8.54 µm under induction conditions (σ = 5.421). In isolates 2257, 2503, 2583, 2619 and 3102, no increase in capsule size was observed (Figure S3).

*Capsular induction—MLST*. When analyzing the capsule sizes with the most frequent STs (*n* = 21 isolates), we observed that ST69 and ST95 presented on average a greater increase in capsule size of 1.938 and 1.624 µm, respectively. Regarding the isolates belonging to ST2 and ST377, smaller changes of 0.347 and 0.425 µm were observed, respectively. A statistically significant association (*p* > 0.002) was found between the most frequent STs (ST2, 69, 97 and 377) and the induction of the capsule induction medium. On the other hand, for the comparison of the capsular sizes of the most frequent STs in the pre- and post-inoculation test in *G. mellonella*, similar results were observed to those obtained in the induction with the capsule induction medium with ST69, ST95 and ST377. In contrast, the results obtained for ST2 differ, since the average capsule size obtained in the invertebrate model was 1.158 µm, and that in the capsule induction medium was 0.347 µm. In these results, no statistically significant association was observed between the induction of capsular growth and the results obtained in pathogenicity (*p* = 0.890) or pre- and post-inoculation capsular size in *G. mellonella* (*p* = 0.283).

### 3.5. Melanin Production

**Figure 5:**
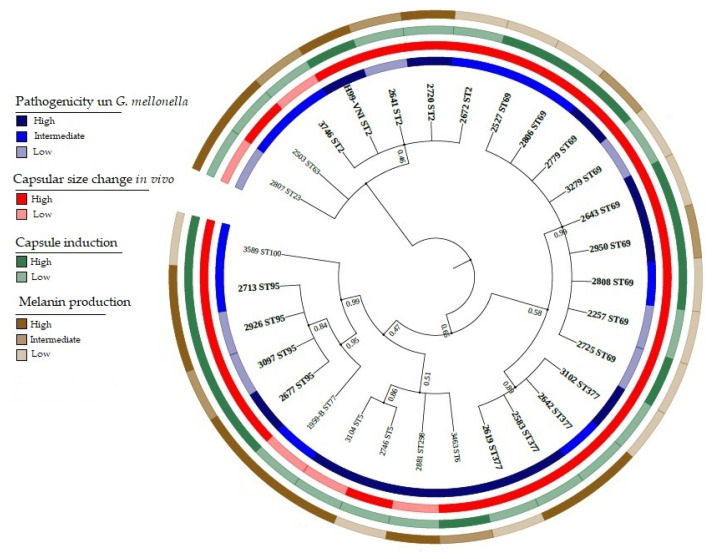
Summary of the main genotypic and phenotypic results obtained in 29 clinical isolates of *C. neoformans* var. *grubii*: Phylogenetic analysis, pathogenicity in *G. mellonella*, capsule size, and melanin production. The evolutionary history was derived using the maximum-likelihood method based on the Jukes–Cantor model using concatenated nucleotide sequences of 7 loci. Bootstrap values are displayed for each branch (1000 repeats).

A total of 12 (41.3%) of the isolates presented low melanin production, six (20.6%) presented medium pigmentation, and 11 (37.9%) presented high pigmentation; these results correspond to three biological replicates. An analysis was conducted to determine whether there was a relationship between the results for melanin and the results for pathogenicity, MLST and capsular size (Figure 5, Table S2).

*Pigmentation—pathogenicity*. Of the seven isolates categorized as having low pathogenicity, three were observed to have low pigmentation, two were observed to have intermediate pigmentation and two were observed to have high pigmentation. Additionally, among the high-pathogenicity isolates, isolates with all levels of pigmentation were observed, i.e., four low, three intermediate and five high. No statistically significant relationship was observed between the pathogenicity of isolates and their level of pigmentation.

*Pigmentation—MLST*. In the most frequent STs, it was observed that the isolates belonging to ST69 and ST95 presented greater pigmentation; ST2 presented low pigmentation; and in the isolates of ST377, no such relationship was detected, with both low and high pigmentation being observed (Figure 5).

The susceptibility of the 29 clinical isolates of *C. neoformans* was determined following the National Committee for Clinical Laboratory Standards NCCLS/CLSI guidelines. The antifungal drugs included the two commonly used azoles (fluconazole and voriconazole) and amphotericin B. All the isolates were susceptible to both amphotericin B and voriconazole. For fluconazole, 75.8% of the isolates were classified as susceptible (MICS ≤ 8 µg/mL), and the remaining 24.2% were categorized as sensitive dose-dependent (SDD) (MICs ≥ 16 ≤32 µg/mL). Susceptibility results were not found to be related to clinical variables, sequence type, capsular size, or melanin production (Table S2).

### 3.6. Principal Component Analysis (PCA) of Phenotypic Variables

With the data obtained in the phenotypic assays (Table S2), associations were made between the variants and the phenotypes identified. Initially, associations were made between each virulence factor and the pathogenicity categories identified in the *G. mellonella* model. In this first approach, no significant statistical associations were identified between virulence factors and pathogenicity. Next, we used principal component analysis (PCA) to determine whether there was an association between pathogenicity and virulence factors. Pathogenicity, capsule size (before and after growth in *G. mellonella*, capsular induction medium and delta data), and the results obtained for melanin production were included in this analysis.

**Figure 6:**
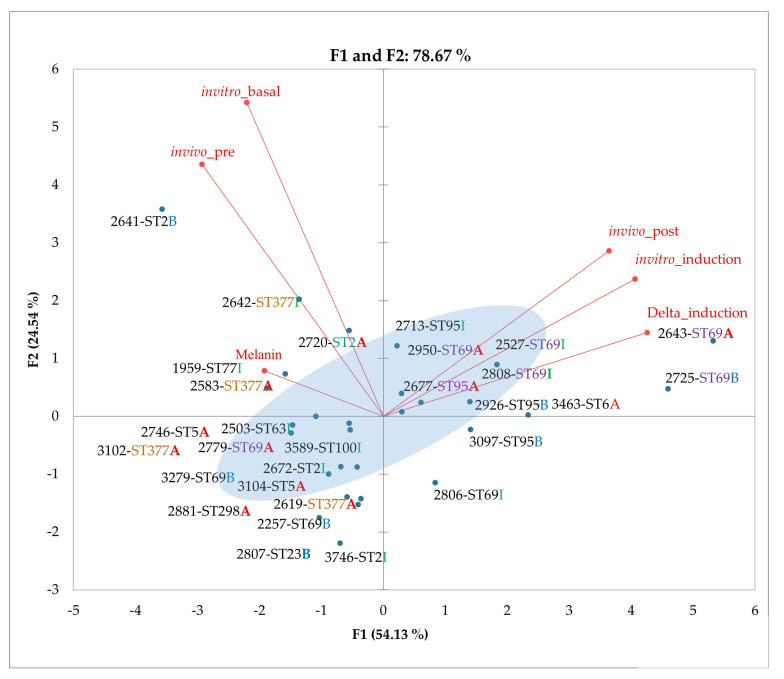
Coordinates calculated via principal component analysis for each isolate and membership of isolates in the proposed categories. A positive correlation is observed; the variable that has the most weight in the analysis is the difference in capsular induction, followed by the variables corresponding to both the in vitro induction in capsular induction medium and the in vivo model. B, I and A correspond to low, intermediate and high pathogenicity, respectively. The modulation of the capsule size can be seen in the blue halo. Filled circles are colored red for active variables and blue for active observations.

In the results obtained by PCA, the greatest contribution of the variables to the first principal component (dimension F1) corresponded to the delta (increase) in capsule size induced by the capsular induction medium, followed by the variables corresponding to both the in vitro induction in capsular induction medium and the post-inoculation in vivo model in *G. mellonella* (Figure 6). Likewise, the variable corresponding to the groups that we proposed to discriminate isolates with high or low capsule size change presented a high contribution in this dimension (see blue halo in Figure 6).

In the second dimension calculated (F2), the variables corresponding to the median initial capsule size presented a greater contribution, both in the pre-inoculation in the in vivo model in *G. mellonella*, and the in vitro basal capsule in the capsule induction medium. The percentage of the variance of data for the first and second dimensions was 54.1% and 24.5%, respectively.

Considering the variation explained for each of the dimensions and the contribution of the variables, the analysis suggests that the variables that measure the modulation of the change in capsule size are the most representative. The isolates can be correspondingly separated into two groups: those with a high change or those with a low change in capsule size. Most isolates were classified as highly pathogenic, regardless of the method used for their evaluation, that is, capsule induction medium or the *G. mellonella* model (Table S2).

### 3.7. Applications of PCA to Phenotypic and Genotypic Variable Sets

A systematic review of the genes that are known or likely to be involved in the main virulence factors or in virulence of *C. neoformans* var. *grubii* was conducted using the Phidias bioinformatics resources (http://www.phidias.us/victors/, accessed on 1 October 2021), the Database of Virulence Factors in Fungal Pathogens (http://sysbio.unl.edu/DFVF/, accessed on 1 October 2021), and the Pathogen–Host Interactions database (http://www.phi-base.org/index.jsp, accessed on 1 October 2021), as well as through bibliographic screening. In total, 322 genes known from the literature to be related to pathogenicity and major virulence factors, capsules and/or melanin were selected or chosen (Table S5).

Briefly, the reads obtained from the complete sequencing of each isolate were mapped against the H99 reference genome using the ReadsAligner module of the NGSEP v4 program. The variants were called with respect to the reference genome sequence using the VCFAnnotate module of NGSEP v4. Finally, only the genomic variants associated with the 322 chosen genes were filtered. To discriminate those variants that have a greater probability of impacting the activity and/or function of the corresponding proteins, the resulting variants were additionally filtered by their location in regions predicted as functional domains using a functional annotation of the reference genome performed using InterProScan.

A total of 5031 biallelic variants of the SNP and indel types were identified, and an additional 21 multiallelic variants were discarded. The identification was carried out in 28 clinical isolates of *C. neoformans* var. *grubii* (MT VNI). Isolate 3589 was discarded from this analysis because it was a single isolate belonging to the phylogenetically distant MT VNII; the large number of single-nucleotide differences between VNI and VNII would have confounded or encumbered analyses of the variation within VNI on which we focus.

Of the total number of variants, 313 corresponded to indels and 4.718 to SNPs. From the latter, 20 were noted as high-impact variants, 964 as moderate-impact, 1.729 as low-impact and 2.318 as modifiers (see Section 2). Variants were selected according to their location in predicted sites, such as functional regions or domains, and 496 non-synonymous variants were considered for further analysis.

These 496 non-synonymous variants from the 322 genes related to virulence factors were then subjected to a principal component analysis to determine the variants with the greatest contribution to the genetic variability observed among clinical isolates. To select the variants with the highest contributions to the first three principal components, the loading values for those three components were calculated. The annotations of the identified genes in the FungiDB database (http://fungidb.org/, accessed on 20 October 2021) were used to provide the descriptions and gene names.

**Figure 7:**
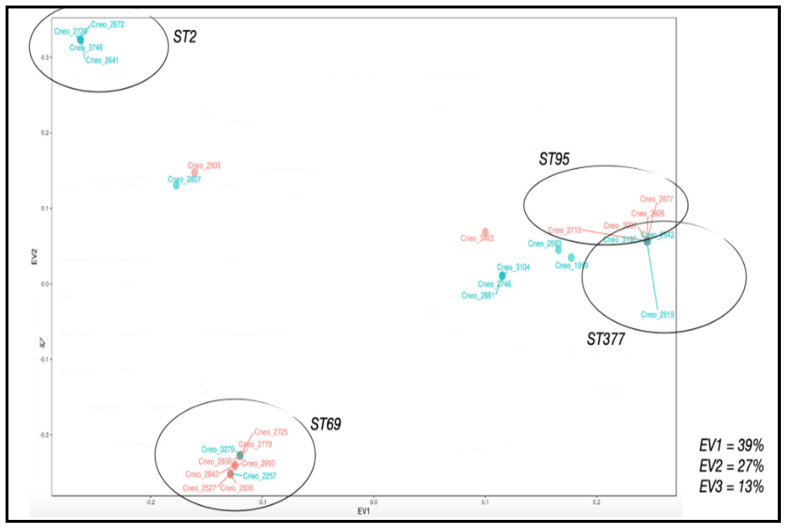
Principal component analysis for the genomic variants of 322 genes related to capsule, melanin, and pathogenicity. The figure shows the coordinates for each of the isolates as well as inclusion in the groups proposed for change in capsule size (red: isolates with high change; blue: with low change). The groups or clusters formed appear in circles.

The percentages of the total variance explained by the first, second and third principal components were 38%, 27% and 13%, respectively. These three components, thus, explain 78% of the variation observed at the genetic level for the 322 genes. The isolates were grouped by PCA into four clusters, within which the isolates could be discriminated by ST (Figure 7). A total of 157 variants were filtered by the contribution to the main components, of which 52 contributed in greater proportion to the first component, 64 variants to the second component and 41 variants to the third main component. These variants were present in 86 of the 322 genes under study.

We determined the genomic variants associated with the groups of isolates proposed for capsular size (high and low), melanin production (high, intermediate, and low), and pathogenicity (high, intermediate, and low). Fisher’s exact test was performed for each of the 496 SNPs using the Fisher test function in R to evaluate the hypothesis of independence between the genotypes and their membership in phenotypic groups. All *p*-values were filtered with a significance value of 0.05.

A total of 48 non-synonymous variants from 39 protein-coding genes were associated with the phenotypes observed and by the proposed groups; of these, 18 SNPs were found to be associated with capsule size phenotypes, 29 with melanin production phenotypes, and 1 with pathogenicity phenotypes (see Figure 5, Table S6).

**Figure 8:**
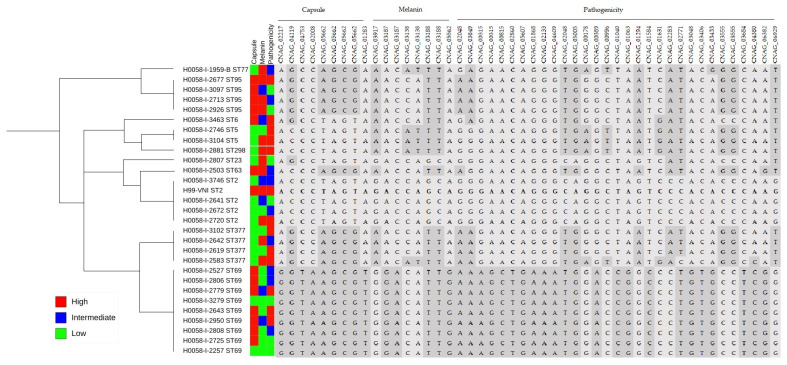
Gene variants associated with phenotypic groups and STs. The reference sequence is in bold (H99). The evolutionary history as inferred from the MLST data was reconstructed via the maximum likelihood method and the Jukes–Cantor model using concatenated nucleotide sequences of 7 loci. From phylogenetic analysis of pathogenicity in *G. mellonella*, capsule size and melanin production, the 39 genes that grouped the 48 non-synonymous variants identified are shown.

The 39 genes that grouped the 48 non-synonymous variants identified are shown in Figure 8. Four genes related to the GO term “pathogenicity” were identified (*PMT4*, *IPK1*, *IRK3* and CNAG_05005), as were four genes related to organization in the external encapsulation structure (*OVA1*, *ROM2*, *PTP1* and CNAG_04753), and three genes related to biosynthetic and metabolic processes of melanin (*SER202*, *NUP75* and *PBX2*) (Tables S5 and S6).

Regarding the STs, only the ST69 exclusive polymorphism was found in the *IRK3* and *PBX2* genes as well as in the four capsule-related genes. This ST was characterized by isolates related to low melanin production, high capsule production, and varied pathogenicity. No association was observed among the results obtained in pathogenicity in *G. mellonella*, or the STs, capsular size, melanin production or nonsense variants evaluated (Figure 8). In the isolates belonging to ST2 in which H99 was found, it was observed that the genes analyzed did not present genetic variability, so these genes could support the differentiation of ST.

## 4. Discussion

The results of this study represent progress toward deciphering the association among pathogenicity, production, and polymorphisms of capsule/melanin in *Cryptococcus neoformans* var. *grubii* VNI, given the high variability of pathogenicity and virulence factors in reference strains.

### 4.1. Genotypic Diversity in a Group of Clinical Isolates of C. neoformans var. grubii by MLST

The genotypes of 29 clinical isolates of *C. neoformans* var. *grubii* of Colombian origin were first characterized by MLST typing. High variability was identified with 11 STs, of which three—ST95, ST298, and ST377—had not been previously reported in Colombia [48]. In addition, four high-frequency sequence types reported globally in clinical isolates were identified, namely, ST2, ST69, ST95, and ST377 [49,50,51]. The results of this study are similar to those described in Mexico by Beale et al., showing high levels of diversity with 50 STs in 230 isolates of *C. neoformans* var. *grubii.* Furthermore, among the STs described as most frequent, two were also most frequently identified in the present study: ST69 and ST93 [49]. It should also be noted that in an Italian study with clinical isolates of *C. neoformans* var. *grubii*, ST69 was the second most prevalent [50]; in contrast, in studies conducted in Indonesia and Kuwait, this sequence was not reported to be frequent [51]. Type ST95 is one of the most frequent in countries including China, India, Indonesia, South Africa, Uganda, Thailand, and Brazil [52,53,54]. For example, in Brazil, ST95 is considered one of the most prevalent genotypes in the north of the country [55]. Another of the most frequent STs found in this study was ST2, which is common to the H99 reference strain and has been sporadically detected in clinical isolates in Colombia [48]. ST2 has been reported with low frequency in other countries, including countries in Africa as well as Argentina, Brazil and the United States of America; conversely, in Germany, there is a high prevalence of this ST [49,56,57].

In a recent study carried out by the Latin American Cryptococcosis Study Group, 41 STs were identified from published data available on *C. neoformans.* The most frequent sequence types encountered among clinical, environmental, and veterinary strains included ST2, ST5, ST23, ST77, and ST93 [58]. These findings are comparable with the present investigation, in which we report a high frequency of ST2 and ST93. These STs are genetically related, and are grouped into clades close to the STs of African and Asian isolates [49,51].

### 4.2. Pathogenicity Is Independent of Characterized Genotype in a Group of Clinical Isolates of C. neoformans var. grubii

The larvae of the wax moth *G. mellonella* constitute a widely used invertebrate model for in vivo studies of *Cryptococcus* pathogenicity and virulence, in which the yeast is able to proliferate in the caterpillar hemocoel [32,33,59]. This invertebrate, in general, has powerful innate immune mechanisms that include the presence of hemocytes with phagocytic activity. The hemocytes resemble the activity of the macrophages and neutrophils of mammalian organisms [59,60].

The determination of pathogenicity in *G. mellonella* showed different profiles among the 29 clinical isolates used. Highly pathogenic isolates killed *G. mellonella* within a range of 2 to 3 days, those of intermediate pathogenicity in 4 to 5 days, and those with low pathogenicity in 6 or more days. Thus, twelve isolates were classified as highly pathogenic, ten as intermediate, and seven as low. The classification was made taking into account results for the reference strain H99, which is considered a highly pathogenic strain [47]. The high observed pathogenicity variability among the isolates was not associated with any of the clinical demographic variables screened, such as sex, age, or geographical origin of the patients in whom the fungus was recovered, nor was it associated with the genotypic classification obtained from the STs.

A similar approach for assessing levels of pathogenicity has been used in several investigations, in which the *G. mellonella* model has made it possible to differentiate more or fewer pathogenic isolates [33]. One of these is a South African study in which the authors observed heterogeneity of pathogenicity in clinical isolates; however, pathogenicity as estimated was not related to patient outcome [61]. Perhaps the pathogenic differences between the isolates are affected by the environmental conditions in which the fungus grows, the model used, and the host conditions. Indeed, in our group, we have results supporting the fact that the bioavailability of metals, especially pre-incubation with copper and iron salts, increases the production of virulence factors and their pathogenicity (data soon to be released).

### 4.3. Classification of Pathogenic Isolates of C. neoformans var. grubii via the Main Virulence Factors: Capsule and Melanin

The capsule and melanin contribute to the pathogenicity of the complex of species that cause cryptococcosis. Different studies have proposed that the interaction with environmental predators, such as amoebas, has led to a selective pressure on this yeast; this is thought to have resulted in characteristics that allow it to be prepared for extreme environments and adapt, so as to have an advantage when infecting mammals [22].

Considering that the capsule is the first component that interacts with the host immune system, its dynamic nature contributes to virulence by allowing the fungus to evade immune defenses in the host [26]. In this work, capsule size was evaluated using two approaches: (a) pre- and post-inoculation in the *G. mellonella* model and (b) a capsule induction medium. Significantly larger capsules were observed after the fungus was inoculated in the larvae, which is consistent with reports on infection in mammals and in this invertebrate, which show that virulence factors are inducible in the infection process in response to immune defense [33]. *In vitro* capsule induction has been described using several approaches, one of which is the use of capsule induction medium 50 mM, pH 7.3, where capsule growth is favored by an alkaline pH [34]. In this medium, similar results to those obtained in the invertebrate model were observed in the increase in the capsule size. Indeed, it was found that measuring capsule change in vivo or in vitro gives comparable results. The occasional differences in capsule size change observed between the two methodologies could be related to characteristics of the host or the environment in which the yeast grows. In fact, changes in environmental conditions such as CO_2_, pH and serum, as well as low iron and high copper concentrations, have been reported to induce capsular enlargement [19,62].

Assessing the amount of melanin in clinical isolates of *C. neoformans* makes it possible to identify strains with a greater capacity to produce this pigment [17,63,64,65,66]. Several studies propose that strains that are not capable of producing melanin exhibit a significant reduction in virulence [67,68]. Here, the pathogenicity profiles of the clinical isolates were not related to melanin production. Renney et al. [69] suggested that the rate of melanization could be more important for virulence than the final amount of melanin, since melanization protects cells against immune mechanisms, and cells that melanize earlier would have a survival advantage. Sabiiti et al. showed that secreted melanin helps the survival of yeast in cerebrospinal fluid, but when they analyzed the amount of melanin produced, they did not observe a statistically significant correlation [70]. Therefore, the melanization rate, rather than the final amount, could be associated with the pathogenicity profiles.

### 4.4. Identification of Non-Synonymous Variants Associated with Capsule and Melanin Genes That Could Influence Pathogenicity

After sequencing the complete genome of the 29 clinical isolates of *C. neoformans* var. *grubii*, we screened the non-synonymous variants in a set of 322 genes that were known or predicted to be related to the main virulence factors, and that could, thus, possibly influence its pathogenicity for possible associations with the phenotypes on which we had focused. A total of 48 nonsynonymous variants were found to be robustly associated with at least one of the observed phenotypes, and were found to be located in the predicted functional domains of 39 of the 322 genes. Some of the 48 SNPs identified are located in known genes related to capsular polysaccharide and/or melanin synthesis (e.g., *PTP1*, *PMT4*, *IPK1*, *PBX2*, *OVA1* and *ROM2*).

The gene encoding the polyol transporter protein *PTP1* (with identifier CNAG_05662 or its synonym *ITR4*) is involved in functions such as proliferation, cell differentiation, programmed cell-death and stress responses [71,72]. In the present investigation, non-synonymous variants were identified in some of the isolates belonging to ST63, ST69, ST77, ST93 and ST377. Furthermore, no association between phenotype and non-synonymy variants in *PTP1* was found. The results are in agreement with those reported by Nielsen et al., who found variants in the *PTP1* gene in 38 clinical isolates of *C. neoformans* classified as ST93 in Uganda. Through logistic regression and principal component analysis, they identified 40 genes, including *PTP1*, a gene that was overexpressed in the intracellular environment of amoebae and murine macrophages. The hypothesis proposed by the authors is that this gene may be associated with human survival and the immune response [73].

Alteration of the *PMT4* gene encoding the dolichyl-phosphate-mannose mannosyltransferase protein in *C. neoformans* affects virulence and growth at 39 °C, and increases sensitivity to amphotericin B [74]. Here, the variant found was identified in four isolates belonging to ST5, ST77 and ST298, which exhibited small capsules. Mutant strains in this gene present morphological defects and cell wall alterations, possibly due to changes in the composition and synthesis of glucans [75]. The protein O-mannosyltransferases (Pmt proteins) play an important role in the secretion, localization and function of many cell wall proteins involved in the synthesis of their components, such as glucan, as well as in the integrity of the cell wall and morphogenesis [76].

The *PBX2* gene encodes the parallel beta-helix repeat protein that acts in cell wall remodeling to maintain cell morphology and the availability of precursors for other synthetic glycan processes [77]. The variant of this gene was identified in the isolates belonging to ST69, which mostly present large capsules and little melanin production. The absence of the *PBX2* gene’s product affects the growth of the cell wall and the capsule. Furthermore, it has been observed in murine models that the absence of this gene results in surface changes that induce further phagocytosis [78].

Inositol polyphosphate kinases (*IPKs*) are interesting targets for antifungal drug design, since mutants that do not produce inositol pyrophosphates exhibit attenuated growth and pathogenicity, compromised cell wall integrity, and reduced melanin and urease production [79]. *IPK* converts the inositol triphosphate product (*IP3*) to inositol pyrophosphates (PP-IP5/IP7) [79]. Here, the *IPK1* variant was identified in 14 isolates belonging to ST5, ST6, ST63, ST77, ST95, ST298 and ST377, most of which were grouped into isolates with high pathogenicity.

*OVA1* is a key member of the genes that encode lipid-binding proteins. It plays an important role in vesicle transport [80,81]. Kronstad et al. suggested that *OVA1* is involved in the inhibition of serine proteases and the regulation of signaling components such as heterotrimeric G proteins [82,83]. The variant of this gene was identified in the isolates belonging to ST69. Hu et al. observed, in 2007, that *OVA1* plays a regulatory role in the trafficking of proteins and polysaccharides to the cell surface of *C. neoformans* as a component of secretory vesicles. The authors concluded that *OVA1* carries a glycosylphosphatidylinositol (GPI) anchor that may serve to bind the protein to the cell membrane or to β-1,6-glucans in the cell wall [83].

The *ROM2* gene, which encodes the Rho guanyl-nucleotide exchange factor, is involved in growth at 37 °C, mating, and maintenance of cell wall stability under osmotic pressures; participates in the organization of actin and microtubules; and is associated with virulence of *C. neoformans* in mice [84]. Mutants in this gene also present defects in mating, growth at elevated temperatures, and alteration in the cell wall (since there is a deficiency of (1,3) β-d glucan) [85]. The latter is related to the fact that Rom2 is involved in the activation of the GTPase Rho1, which activates the protein kinase Pkc1, involved in cell wall integrity [86,87]. Here, the variant of this gene was identified in 20 of the Colombian isolates belonging to ST6, ST23, ST69, ST77, ST95 and ST377, most of which were characterized by large capsules. It would be interesting to further explore this phenomenon to determine whether there is any relationship between the *ROM2* variant detected, this capsular phenotype, and the cell wall of these clinical isolates.

When we analyzed the non-synonymous variants according to the identified sequence type, we observed that the isolates belonging to ST69 presented more SNPs than the isolates of the other frequent STs (ST2, ST95 and ST377). The ST69 isolates were the only ones that presented a nucleotide change in 19 genes, including *SIT1*, *EBG1*, *CAP5*, *SOL3*, *AGO1*, *OVA1*, *CHS7*, *IRK3* and *SPP101* (see Table S5). Furthermore, ST69 was characterized by low melanin production and large capsules in most isolates. Mukaremera et al. [88] recently proposed that in vivo studies of closely related clinical strains, such as those having identical STs, should be combined with genomic analyses to facilitate the identification of novel genes that are critical in vivo. The authors demonstrated that infections with *C. neoformans* strains of the same ST by MLST could have different clinical and phenotypic outcomes. Other investigations affirm that there may sometimes be a relationship between STs and clinical outcomes [89,90].

## 5. Conclusions

The Colombian clinical isolates of *C. neoformans* var. *grubii* showed different levels of pathogenicity, which are multifactorial and susceptible to modulation. The pathogenicity and virulence factors evaluated were not associated with a particular ST in the clinical isolates analyzed. The combination of individual characteristics that reflect different phenotypic profiles for each isolate was therefore studied.

ST69 was the most frequent of the clinical isolates. The isolates that are present in this ST were characterized by high pathogenicity, small capsules, and, in most cases, low melanin production. Our principal component analysis suggests that, at least in the isolates studied, capsule size can be differentiated, with results that appear to be largely independent of the method used for induction.

## Data Availability

All experimental data are provided in the manuscript and in supplementary files or are available via the NCBI BioProject database, with the accession number PRJNA669191.

## Supplementary Materials

The following supporting information can be downloaded at: https://www.mdpi.com/article/10.3390/jof8030245/s1, Figure S1: Flow chart of the approaches used to identify *C. neoformans* genes associated with observed phenotypes. Principal component analysis (PCA) was used. Variables obtained from the main virulence factors (Capsule and melanin), associated genes and pathogenicity obtained from the *G. mellonella* model; Figure S2: Capsular size in 29 clinical isolates of *C. neoformans* var. *grubii*. Results obtained from the capsular size pre and post-inoculation in *G. mellonella*. *p* < 0.001 (***); *p* < 0.0001 (****); Figure S3: Results of the induction of capsule growth in MOPS in 29 clinical isolates of *C. neoformans* var. *grubii*. *p* < 0.05 (*); *p* < 0.01 (**); *p* < 0.001 (***); Table S1: Demographic variables of isolates; Table S2: Results of pathogenicity, capsule, melanin, susceptibility, MLST and demographic variables of 29 clinical isolates of *C. neoformans* var. *grubii*; Table S3: MLST allelic profiles for 29 clinical isolates of *C. neoformans* var. *grubii*. One MT-VNII isolate is shown in gray; Table S4: Classification of the pathogenicity of clinical isolates of *C. neoformans* according to results obtained in the invertebrate model *G. mellonella*, and reference strains with high (H99) and low (JEC21) pathogenicity; Table S5: List of 322 genes associated with pathogenicity, capsule, and melanin in *C. neoformans*; Table S6: Genomic variants associated with phenotypic groups and sequence types. Refs. [91,92] are cited in Supplementary Materials.
