# Supplementary material for: Deciphering the Association among Pathogenicity, Production and Polymorphisms of Capsule/Melanin in Clinical Isolates of Cryptococcus neoformans var. grubii VNI
Source: J Fungi (Basel). 2022 Feb 28;8(3):245. doi: 10.3390/jof8030245 (PMC8950468; doi:10.3390/jof8030245)
Supplement: Supplementary file 1 [file jof-08-00245-s001.zip › jof-1546291-supplementary/jof-1546291-supplementary.pdf]

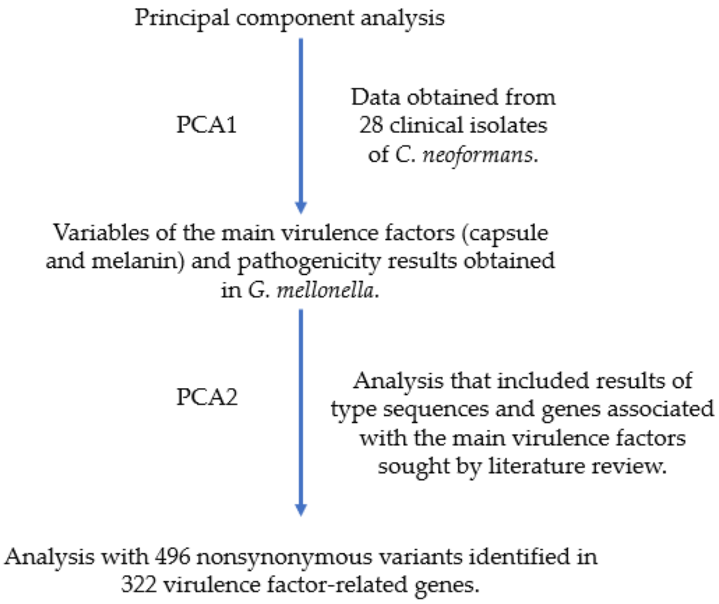

Figure S1. Flow chart of the approaches used to identify *C. neoformans* genes associated with observed phenotypes. Principal component analysis (PCA) was used. Variables obtained from the main virulence factors (Capsule and melanin), associated genes and pathogenicity obtained from the *G. mellonella* model.

Principal component analysis (PCA) provides a multivariate generalization of the bivariate orthogonal regression (major axis regression[1]) for two-dimensional scatterplots (biplots). Where two variables x and y are correlated, the more traditional linear regression determines a straight line that minimizes the vertical (y, dependent variable) distances of the points from the line. By contrast, the orthogonal regression (PCA in 2 dimensions) proceeds by calculating a straight line (major axis, representing the first principal component, PC1 or F1) that minimizes the points' orthogonal distances from the line.

The generalization to n (> 2) variables seeks to obtain the similarly constructed principal components PC1, PC2, …, PCn in an n-dimensional space[2]. Often a large part of the variability in the n-dimensional cloud of points can be well represented by just the first two or three principal components, so that for example a scatterplot of PC2 versus PC1 can already serve as a biologically informative characterization of the variation in the full multivariate data set. Each principal component is a linear combination (weighted or loaded sum) of the original empirical variables, and those original variables' contributions to PC1 and PC2 can often already reveal their most important roles or relationships.

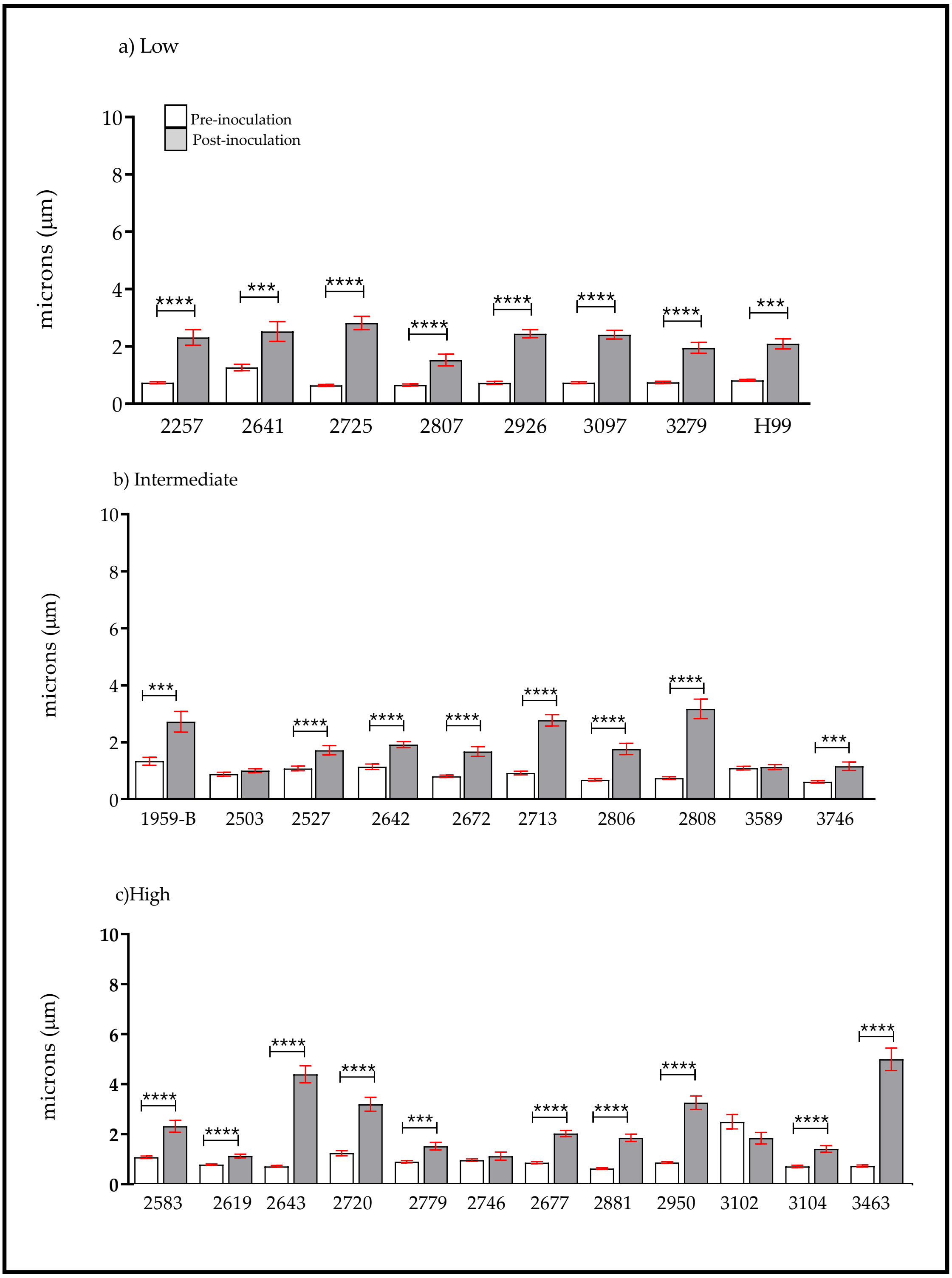

Figure S2. Capsular size in 29 clinical isolates of *C. neoformans* var. *grubii.* Results obtained from the capsular size pre and post-inoculation in *G. mellonella*. p <0.001 (***); p <0.0001 (****).

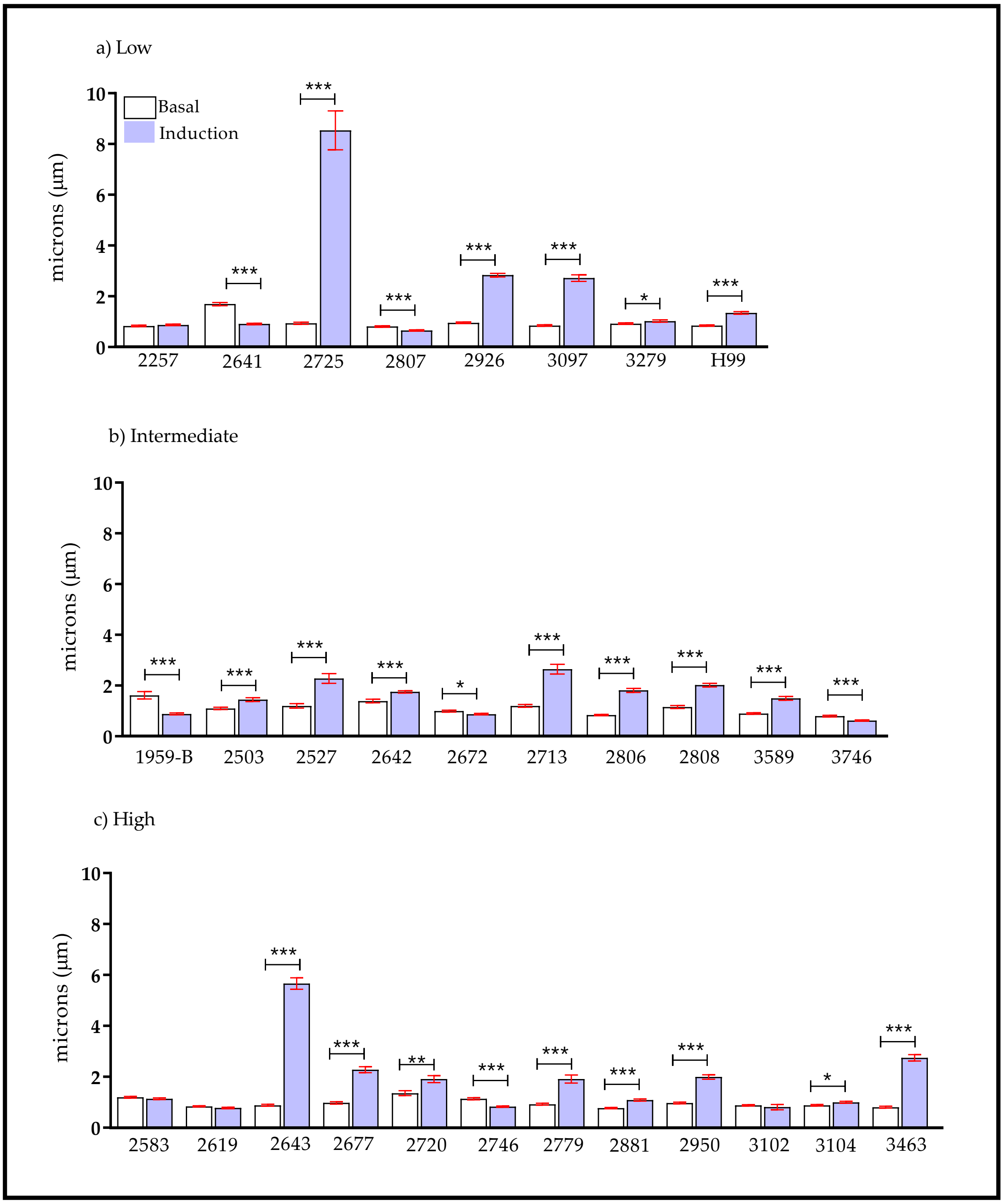

Figure S3. Results of the induction of capsule growth in MOPS in 29 clinical isolates of *C. neoformans* var. *grubii*. $p < 0.05$ (*); $p < 0.01$ (**); $p < 0.001$ (***).
